# Draft Genome Sequence of Enterococcus faecalis UMB1309, Isolated from Catheterized Urine

**DOI:** 10.1128/MRA.00406-20

**Published:** 2020-05-28

**Authors:** Adam Schwartz, Taylor Miller-Ensminger, Adelina Voukadinova, Alan J. Wolfe, Catherine Putonti

**Affiliations:** a Department of Biology, Loyola University Chicago, Chicago, Illinois, USA; b Bioinformatics Program, Loyola University Chicago, Chicago, Illinois, USA; c Department of Microbiology and Immunology, Stritch School of Medicine, Loyola University Chicago, Maywood, Illinois, USA; d Department of Computer Science, Loyola University Chicago, Chicago, Illinois, USA; University of Arizona

## Abstract

A strain of Enterococcus faecalis was isolated from catheterized urine. Here, we present the draft genome sequence of this isolate, E. faecalis UMB1309. Analysis of the genome revealed multiple genes coding for virulence factors, as well as genes associated with antibiotic resistance.

## ANNOUNCEMENT

Enterococcus faecalis has long been identified as a commensal organism found in the gastrointestinal tract of healthy individuals and as a low-grade opportunistic pathogen (1–3) associated with endocarditis, urinary tract infections (UTIs), sepsis, bacteremia, and meningitis (2–4). Over the past 50 years, however, the emergence of multidrug-resistant strains of enterococci has contributed to its rise to become the third leading cause of nosocomial infections today (1–5). Of all nosocomial infections caused by E. faecalis, UTIs are the most common (3, 6). Here, we present the draft genome of E. faecalis UMB1309, which was isolated from the catheterized urine of a woman with a UTI.

E. faecalis UMB1309 was isolated from a catheterized urine specimen from a woman seeking clinical care at the Loyola University Medical Center Female Pelvic Medicine and Reconstructive Surgery Center (Maywood, IL, USA) as part of a previous institutional review board (IRB)-approved study (7). This strain was isolated using the expanded quantitative urinary culture (EQUC) protocol (8). Matrix-assisted laser desorption ionization–time of flight (MALDI-TOF) mass spectrometry was used to determine the genus and species of the isolate following a previously published protocol (8). The isolate was then stored at −80°C. The isolate was streaked onto a Columbia nalidixic acid (CNA) agar plate and incubated at 35°C with 5% CO_2_ for 24 h. A single colony was selected and added to liquid brain heart infusion (BHI) medium, which was then incubated again under the aforementioned conditions. DNA was extracted using a Qiagen DNeasy blood and tissue kit following the manufacturer’s protocol for Gram-positive bacteria with the following modifications: we used 230 μl of lysis buffer (180 μl of 20 mM Tris-Cl, 2 mM sodium EDTA, and 1.2% Triton X-100 and 50 μl of lysozyme) in step 2 and altered the incubation time in step 5 to 10 min. DNA was quantified using a Qubit fluorometer. The DNA was sequenced at the Microbial Genomic Sequencing Center at the University of Pittsburgh. DNA was enzymatically fragmented using an Illumina tagmentation enzyme, and then indices were attached using PCR. The DNA library was sequenced using a NextSeq 550 flow cell; this produced 2,145,494 pairs of 150-bp reads. Raw reads were quality controlled by trimming using Sickle v1.33 (https://github.com/najoshi/sickle). The genome was then assembled using SPAdes v3.13.0 (9) with the only-assembler option for k values of 55, 77, 99, and 127 and annotated using PATRIC v3.6.3 (10). The publicly available genome was annotated using the NCBI Prokaryotic Genome Annotation Pipeline (PGAP) v4.11 (11). The genome coverage was calculated using BBMap v38.47 (https://sourceforge.net/projects/bbmap). Unless otherwise noted, default parameters were used for each software tool.

The E. faecalis UMB1309 draft genome is 3,046,929 bp assembled in 32 contigs and has a GC content of 37.20%. The genome coverage was 173×, and the *N*_50_ score was 283,553 bp. The PGAP annotation identified 2,892 protein-coding genes, 55 tRNAs, and 5 complete rRNAs (3 5S rRNAs, 1 16S rRNA, and 1 23S rRNA). PATRIC annotation included multiple putative virulence factors, including collagen-binding adhesins, PrgB (associated with cellular aggregation), and other cell wall surface anchor proteins. PATRIC also identified genes conferring antibiotic resistance, including *lsa*(A) (associated with macrolide resistance) and *tet*(M) (associated with tetracycline resistance). Despite the prevalence of E. faecalis infections, the role of this bacterium in the urinary tract is not well understood (4, 6). Further genomic analysis of E. faecalis can reveal insights into these mechanisms and ultimately aid in the development of effective treatments against E. faecalis UTIs.

### Data availability.

This whole-genome shotgun project has been deposited in DDBJ/ENA/GenBank under accession no. JAAUWG000000000. The version described in this paper is the first version (accession no. JAAUWG010000000). The raw sequencing reads have been deposited in the SRA under accession no. SRR11441014.
